# 1,1‐Ethenediol: The Long Elusive Enol of Acetic Acid

**DOI:** 10.1002/anie.201915646

**Published:** 2020-02-12

**Authors:** Artur Mardyukov, André K. Eckhardt, Peter R. Schreiner

**Affiliations:** ^1^ Institute of Organic Chemistry Justus Liebig University Heinrich-Buff-Ring 17 35392 Giessen Germany

**Keywords:** enol, matrix isolation, photochemistry, prebiotic chemistry

## Abstract

We present the first spectroscopic identification of hitherto unknown 1,1‐ethenediol, the enol tautomer of acetic acid. The title compound was generated in the gas phase through flash vacuum pyrolysis of malonic acid at 400 °C. The pyrolysis products were subsequently trapped in argon matrices at 10 K and characterized spectroscopically by means of IR and UV/Vis spectroscopy together with matching its spectral data with computations at the CCSD(T)/cc‐pCVTZ and B3LYP/6–311++G(2d,2p) levels of theory. Upon photolysis at *λ*=254 nm, the enol rearranges to acetic acid and ketene.

## Graphical abstract

**Finally made**: The hitherto unreported 1,1‐ethenediol, the higher energy tautomer of acetic acid, was synthesized and spectroscopically characterized. It can be readily prepared through thermal decarboxylation of malonic acid, as has generally often been implied in the synthetically highly valuable reactions of 1,3‐dicarboxylic acids.

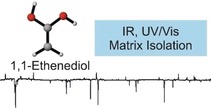

While enols of aldehydes and ketones are commonplace,^1^, ^2^ there is no spectroscopic evidence for the formation of enols from simple aliphatic carboxylic acids, even though the involvement of such enols as primary decarboxylation products of β‐keto acids is common textbook knowledge. Thus far, limited evidence for the existence of acid enols has been provided only for very few examples of conjugated derivatives including benzofulvene‐8,8‐diol,^3^ fulvene‐6,6‐diol,^4^ fluorene‐9‐carboxylic acid enol,^5^ and 2‐phenyl‐1,1,2‐ethenetriol,^6^ in solution by transient time‐resolved UV spectroscopy, which was employed for kinetic studies. O'Neill and Hegarty generated Ar_2_C=C(OH)_2_ enols with Ar=mesityl and pentamethylphenyl^7^, ^8^, ^9^ through hydration of the corresponding ketenes.

Frey and Rappoport demonstrated the generation of kinetically stabilized but short‐lived enols (Tip_2_=C(OH)_2_, Tip_2_=C(OH)NMe_2_, tip=2,4,6‐triisopropylphenyl) using bulky substituents by addition of water and dimethylamine to the corresponding ketene; the enols were observed and analyzed by NMR spectroscopy.^10^, ^11^ The p*K*_Enol_ values were experimentally estimated^6^ and computed;^12^, ^13^ these are much higher than those of aldehydes and ketones. The p*K*_Enol_ value for the parent CH_3_COOH/CH_2_=C(OH)_2_ pair was estimated to be in the range of 18–21^14^, ^15^ in reasonable agreement with the computed values of 19–25.^12^, ^16^, ^17^ Enols of carboxylic acids have been intensively studied computationally and found to be substantially less stable than their acid tautomers.^18^, ^19^, ^20^

Spectroscopic data for the parent aliphatic acid enol, 1,1‐ethenediol (**1**), the enol of acetic acid (**3**) have not been reported (Scheme 1). As enol formation through decarboxylation reactions are key synthetic (as often found in the post‐hydrolysis steps of Knoevenagel‐type reactions)^21^ and a variety of biologically relevant reactions (such as enzymatic decarboxylation),^22^ direct spectroscopic information of such species is highly valuable for their identification in such processes and for understanding their stabilities and reactivities.

**Scheme 1:**
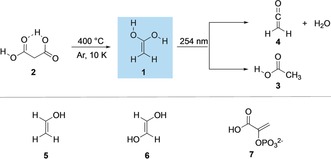
1,1‐Ethenediol (**1**) generated from malonic acid (**2**) through pyrolysis and trapping in an argon matrix. Subsequent photorearrangement to acetic acid (**3**) and ketene (**4**). Biologically highly relevant enols (**5**–**7**).

More generally speaking, most enols are short‐lived reactive species and they readily isomerize to their keto‐forms; enols can be stabilized through resonance,^23^ steric factors,^1^ and electronic effects.^2^, ^24^ Experimental methods such as time‐resolved spectroscopy,^5^, ^25^ neutralization‐reionization mass spectrometry,^26^, ^27^ NMR as well as IR spectroscopy,^28^ and other spectroscopic techniques^29^ along with computational methods^30^, ^31^, ^32^, ^33^ have played a significant role in understanding the structures and reactivities of enols. The simplest enol, ethenol (vinyl alcohol), was generated in the gas phase through the pyrolysis of ethylene glycol.^29^ Schauermann et al. reported the synthesis of the enol of acetophenone on metal surfaces stabilized by intermolecular interactions with the keto form of a second molecule to give a ketone‐enol dimer.^34^ Turecek et al.^26^ generated the enol of γ‐butyrolactone in the gas phase and characterized it by neutralization–reionization mass spectrometry. They found that the lactone enol persists as an isolated molecule in the gas phase.

The potential role enols play in the chemistry of the interstellar medium has been implied with ethenol (**5**) that was first detected through microwave emissions from Sagittarius B2 in 2001.^35^ Several enols were detected in cold plasma discharges of alcohols, suggesting that enols can form from alcohols (commonly available interstellar species) by ultraviolet and cosmic radiation.^36^ 1,2‐Ethenediol (**6**), the enol of glycolaldehyde, has been suggested to contribute to the formation of carbohydrates (pentoses) in early Earth environments.^37^ Recently, Coggins and Powner^38^ demonstrated the synthesis of phosphoenol pyruvate (**7**), a high energy phosphate found in living organisms, from prebiotic precursors such as glycolaldehyde and glyceraldehyde. Enols have been suggested as important reactive intermediates for the formation of secondary organic aerosols in the atmosphere,^39^, ^40^, ^41^, ^42^, ^43^ as enols can readily be oxidized to carboxylic acids in the gas phase.^41^, ^43^, ^44^

The fragmentation of molecular precursors presents a suitable entry point to explore the synthesis of enols in the gas phase. As established historically, dicarboxylic acids are attractive enol precursors due to their facile preparation, their tendency to undergo thermal decarboxylation under mild conditions, and their release of an inert and neutral byproduct (Scheme 1).^45^ Herein, we report the synthesis of **1** and its deuterated analogue (*d*_4_‐**1**) by decarboxylation of malonic acid (**2**) and *d*_4_‐malonic acid (*d*_4_‐**2**) through flash vacuum pyrolysis (FVP) at 400 °C and capture of the pyrolysis products in an argon matrix at 10 K. We characterized **1** and *d*_4_‐**1** by measuring their IR spectra and comparing them with IR bands computed at AE‐CCSD(T)/cc‐pCVTZ + ZPVE level of theory.

Inspection of the IR bands of an IR spectrum recorded after pyrolysis at 400 °C (Figure 1 and Figure S1 in the Supporting Information) demonstrates remarkably good agreement between the measured and computed (AE‐CCSD(T)/cc‐pCVTZ) vibrational bands, which provides convincing evidence for the successful preparation of **1**. In particular, the strong band at 1712 cm^−1^ can be assigned to the C=C stretching mode; matrix site effects cause splitting of this band. Other strong bands at 3643 and 3619 cm^−1^ can be assigned to the OH stretching modes of **1** (Supporting Information, Figure S2). The bands at 730 and 674 cm^−1^ can be attributed to the wagging and rocking modes of the methylene group of **1**. With the help of the computations, other strong and medium intensity bands were unambiguously assigned to **1** (Supporting Information, Table S1). In addition to **1**, the matrix contains a large amount of CO_2_, indicating nearly complete consumption of **2**, some typical impurities (for example H_2_O) and acetic acid (**3**) formally derived from **1** via a 1,3‐[H] migration and ketene (**4**). The assignments of these bands were also confirmed with a *d*_4_‐**1** labelled derivative that leads to characteristic isotopic shifts. For example, the intense IR band at 1712 cm^−1^ shows a red shift of −44 cm^−1^ (calc.: −57 cm^−1^) in *d*_4_‐**1**. The OH stretching modes at 3643 and 3619 cm^−1^ are red‐shifted by −954 and −944 cm^−1^ (calc.: −1049 and −1045 cm^−1^) in *d*_4_‐**1**. Overall, the good agreement between the computed (AE‐CCSD(T)/cc‐pCVTZ) and experimentally measured frequencies of the **1** and *d*_4_‐**1** isotopologues underlines the successful preparation of this elusive species (Figure 1, and Table S1 in the Supporting Information).

**Figure 1:**
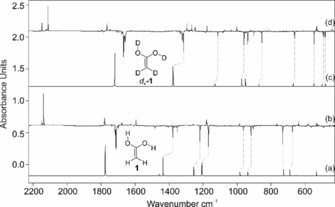
IR spectra showing the pyrolysis product of **2** with subsequent trapping in an argon matrix at 10 K. a) IR spectrum of **1** computed at AE‐CCSD(T)/cc‐pCVTZ (unscaled). b) IR difference spectra showing the photochemistry of **1** after irradiation with *λ*=254 nm in argon at 10 K. Downward bands assigned to **1** disappear while upward bands assigned to **3** and **4** appear after 20 min irradiation time. c) IR spectrum of *d*_4_‐**1** computed at AE‐CCSD(T)/cc‐pCVTZ (unscaled). d) IR difference spectra showing the photochemistry of *d*_4_‐**1** after irradiation with *λ*=254 nm in argon at 10 K. Bands pointing downwards assigned to *d*_4_‐**1** disappear and bands pointing upwards assigned *d*_4_‐**3** and *d*_4_‐**4** appear after 20 min irradiation time.

The molecular structures and thermodynamic properties of **1** and its conformers were studied computationally at various theoretical levels.^12^, ^19^, ^46^ According to these computations, **1** can exist in three distinct conformations with *s‐cis* and *s‐trans* orientations of the OH groups relative to the opposing C−O bond: *s‐cis*, *s‐trans* **1 ct**, *s‐trans*, *s‐trans* **1 tt**, and *s‐cis*, *s‐cis* **1 cc**. At the AE‐CCSD(T)/cc‐pCVTZ level employed here, the most stable conformer is **1 ct** that displays *C*_s_ symmetry and has an ^1^A′ electronic ground state (Figure 2). Conformer **1 tt** is 1.1 kcal mol^−1^ less stable (including the zero‐point vibrational energy correction, ZPVE, denoted as Δ*H*_0_) and shows *C*_2*v*_ symmetry with an ^1^A_1_ electronic ground state. The activation barrier for the **1 ct** → **1 tt** conformational isomerization is only +4.1 kcal mol^−1^. The **1 cc** conformer is the least stable conformer, with a relative energy of 2.8 kcal mol^−1^ above **1 ct**. By comparing the experimentally measured IR spectrum with the computed spectra of **1 ct**, **1 tt**, and **1 cc**, we conclude that indeed conformer **1 ct** preferentially forms in the decarboxylation of **2**, while there is no indication for the formation **1 tt** or **1 cc** directly from pyrolysis.

**Figure 2:**
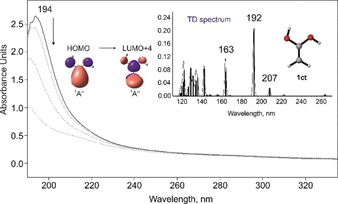
Solid line: UV/Vis spectrum of **1** isolated at 10 K in Ar. Dashed lines: the photochemistry of **1** after irradiation at *λ*=254 nm for 1, 5, and 15 min in argon at 10 K. Inset: computed [TD‐B3LYP/6–311++G(2d,2p)] electronic transitions for **1**.

Irradiation of the pyrolysis products in argon at 254 nm results in complete bleaching of the infrared bands of **1 ct** and the formation of new IR bands. Two distinct species formed simultaneously during photolysis. A new set of IR bands (Figure 1) can readily be assigned to **3 t** by comparison of the IR spectrum with that of an authentic matrix‐isolated sample;^47^ **3 t** forms through 1,3[H] migration. Along with the IR bands of **3 t**, UV irradiation (*λ*=254 nm) of matrix‐isolated **1 ct** resulted in the formation of a strong band at 2138 cm^−1^ with an isotopic shift of −27 cm^−1^ (calc.: −24 cm^−1^ in *d*_2_‐**4**) that can readily be assigned to the C=O stretching mode of ketene (**4**) as the dehydration product of **1**.^48^

Along with the IR analysis, we performed UV/Vis experiments for the identification of **1 ct** (Figure 2). The matrix‐isolated UV/Vis spectrum of **1 ct** exhibits a strong absorption band at *λ*_max_=194 nm. In line with the IR experiments, the transition at 194 nm vanishes upon irradiation (*λ*=254 nm). The strong band at 194 nm correlates well with the computed value at 192 nm (*f*=0.206) at the B3LYP/6–311++G(2d,2p) level of theory. Based on the orbitals involved, the strong band at 194 nm corresponds to a π → π* transition (Figure 2).

The potential energy surface (PES) for the isomerizations of **1 ct** was explored computationally at the AE‐CCSD(T)/cc‐pVTZ + ZPVE level of theory (Figure 3). There are two main pathways through which the isomerizations of **1 ct** can take place. The first reaction path involves the intramolecular [1,3]H‐shift (keto‐enol tautomerization) from one of the hydroxyl groups to the methylene carbon atom to give **3** with a barrier (**TS3**) of 44.6 kcal mol^−1^. A second pathway describes the formation of **4** and water from **1 ct** through a barrier (**TS4**) of 43.9 kcal mol^−1^. Both **3 t** and **4** were observed in our photolysis experiments. The intrinsic reactions paths associated with the rearrangement of **1** to **3** and **4** are almost isoenergetic, with the barrier for the formation of **3 c** being only 0.7 kcal mol^−1^ higher. The fact that we did observe characteristic peaks for **3 t** and **4** directly after FVP suggests that the energy is sufficient in the pyrolysis zone for **1 ct** to undergo a [1,3]H‐shift to **3 t** or to eliminate water to produce **4** (Supporting Information, Figure S1), and that the computations represent well the experimentally found products .

**Figure 3:**
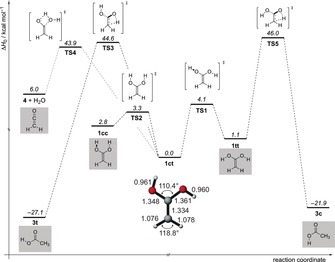
Potential energy profile (Δ*H*_0_) in kcal mol^−1^ of the reactions of enol **1** at AE‐CCSD(T)/cc‐pCVTZ + ZPVE.

In contrast to hydroxycarbenes,^49^, ^50^ quantum mechanical hydrogen tunnelling (QMT) of **1 ct** to **3 t** can be excluded, since we found no evidence for an increase in the bands of **3 t** even after three days in the dark. The matrix spectrum of **1 ct** remained unchanged because the associated barrier is simply too high and too wide to allow hydrogen tunneling. Indeed, the [1,3]H‐shift is associated with a very long computed QMT half‐live of several millions years and can therefore be excluded.

In line with earlier proposals (see above), the above findings bear implications for the chemistry associated with extraterrestrial or prebiotic earth environments. Our results suggest that **1** should be considered as a detectable, new interstellar gas‐phase molecule. The formation of **1** might occur through the exothermic (by 6 kcal mol^−1^, Figure 3) reaction of ketene, which was observed in interstellar clouds,^51^ with water in ice grains. Hydration of ketenes in solution ultimately leading to the corresponding carboxylic acid is a well‐known reaction. The computed energy barrier to this hydration of approximately 38 kcal mol^−1^ is prohibitively high under interstellar conditions. However, it might occur through surface catalysis on ice (for example, through additional water molecules that can significantly lower the barrier for such processes)^52^, ^53^ or in dust grains of molecular clouds. In contrast to acetic acid, which has been spectroscopically identified in molecular gas clouds),^54^ **1** can be considered the more reactive higher energy tautomer of acetic acid that may be involved in the synthesis of biologically relevant molecules. Further study of **1** will be undertaken to fully understand its reactivity and role in prebiotic chemistry.

## Conflict of interest

The authors declare no conflict of interest.

## Supporting information

As a service to our authors and readers, this journal provides supporting information supplied by the authors. Such materials are peer reviewed and may be re‐organized for online delivery, but are not copy‐edited or typeset. Technical support issues arising from supporting information (other than missing files) should be addressed to the authors.

Supplementary
