# Supplementary material for: 1,1‐Ethenediol: The Long Elusive Enol of Acetic Acid
Source: Angew Chem Int Ed Engl. 2020 Feb 12;59(14):5577–80. doi: 10.1002/anie.201915646 (PMC7154680; doi:10.1002/anie.201915646)
Supplement: Supplementary file 1 — Supplementary [file ANIE-59-5577-s001.pdf]

Angewandte Chemie
Eine Zeitschrift der Gesellschaft Deutscher Chemiker

Supporting Information

1,1-Ethenediol: The Long Elusive Enol of Acetic Acid

Artur Mardyukov, André K. Eckhardt, and Peter R. Schreiner**

anie_201915646_sm_miscellaneous_information.pdf

Table of Contents

Experimental Procedures 2
Figure S1 3
Figure S2. 4
Table S1. 5
References 5
AE-CCSD(T)/cc-pCVTZ optimized structures 6

Experimental Procedures

Matrix Apparatus Design. For the matrix isolation studies, we used an APD Cryogenics HC-2 cryostat with a closed-cycle refrigerator system, equipped with an inner CsI window for IR measurements. Spectra were recorded with a Bruker IFS 55 FT-IR spectrometer with a spectral range of 4500−400 cm^{-1} and a resolution of 0.7 cm^{-1} and UV/Vis spectra were recorded with a JASCO V-670 spectrophotometer equipped with an inner sapphire window. A high-pressure mercury lamp (HBO 200, Osram) with a monochromator (Bausch & Lomb) was used for irradiation.

For the combination of high-vacuum flash pyrolysis with matrix isolation, we employed a small, home-built, water-cooled oven, which was directly connected to the vacuum shroud of the cryostat. The pyrolysis zone consisted of an empty quartz tube with an inner diameter of 8 mm, which was resistively heated over a length of 50 mm by a coaxial wire. The temperature was monitored with a NiCr−Ni thermocouple. Malonic acid **2** (Sigma-Aldrich) were evaporated (**2**: 70 °C) from a storage bulb into the quartz pyrolysis tube. At a distance of approximately 50 mm, all pyrolysis products were co-condensed with a large excess of argon (typically 60−120 mbar from a 2000 mL storage bulb) on the surface of the matrix window at 10 K (20 K). Several experiments with pyrolysis temperatures ranging from 200 to 700 °C were performed in order to determine the optimal pyrolysis conditions.

Computations. All coupled cluster computations were carried out with the CFOUR[1] program package. In general, the all electron coupled cluster level of theory[2] including single, double, and perturbatively included triple excitations [AE-CCSD(T)] utilizing the Dunning correlation consistent split valence basis set cc-pCVTZ[3] was employed for geometry optimizations and frequency computations. For all B3LYP[4] computations we used the Gaussian16[5] program package.

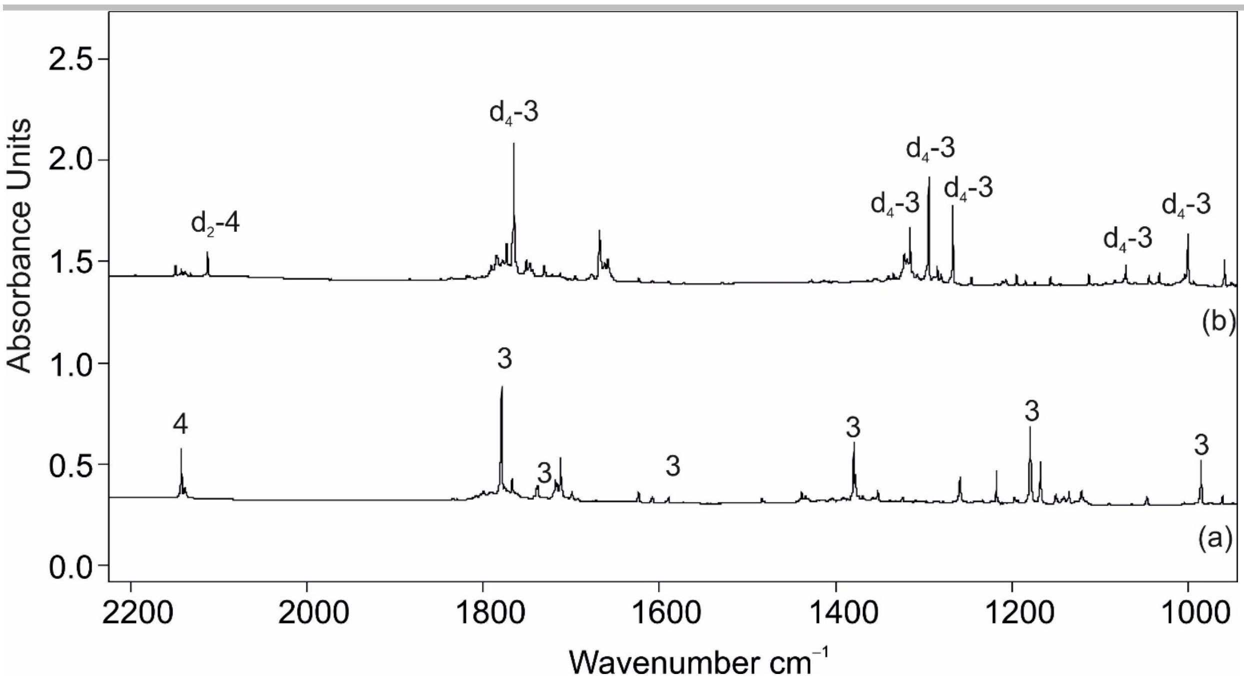

Figure S1. (a) IR spectra showing the product of pyrolysis of **2** in argon matrix with subsequent trapping in an argon matrix at 10 K. (b) IR spectra showing the product of pyrolysis of *d*$_4$-**2** in argon matrix with subsequent trapping in an argon matrix at 10 K.

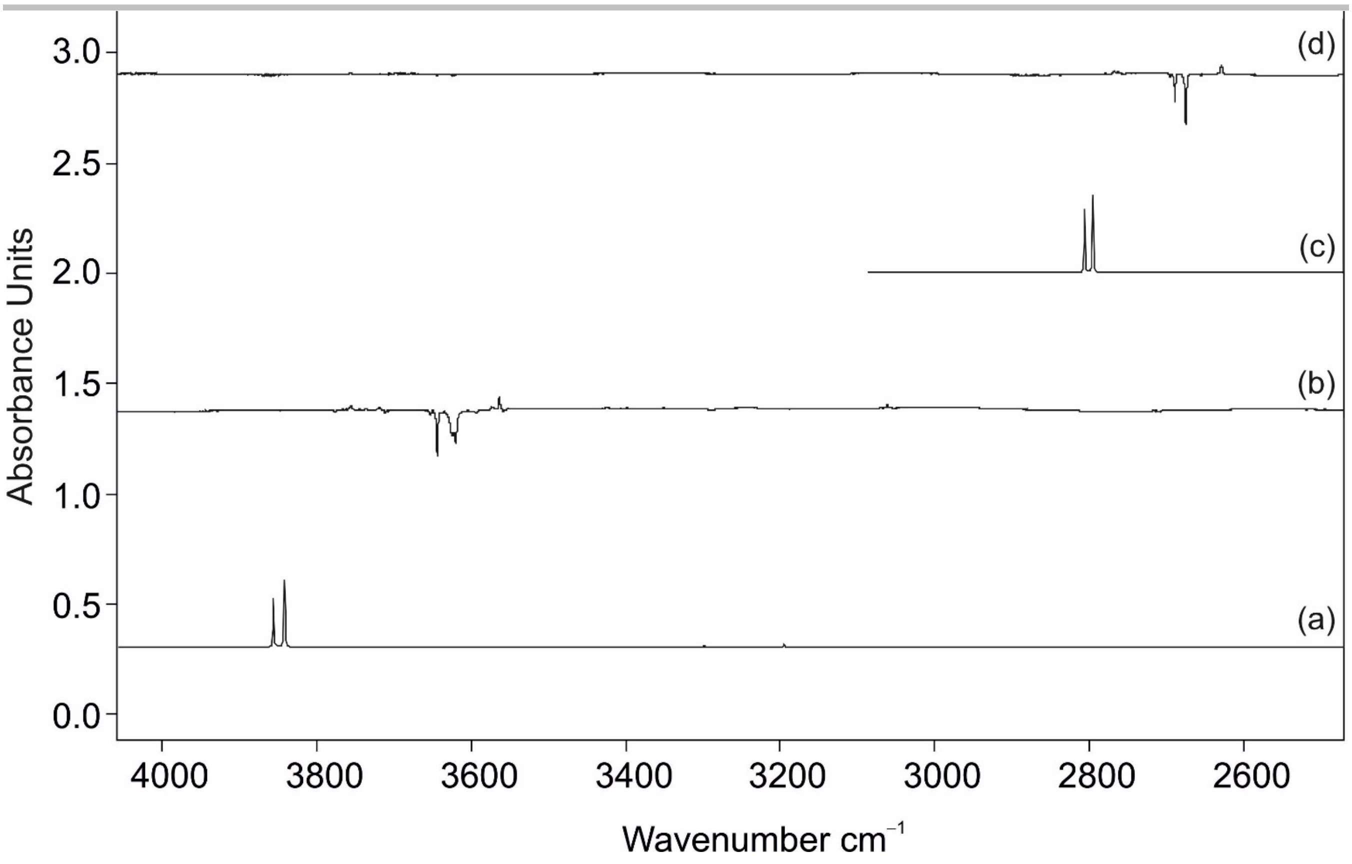

Figure S2. IR spectra showing the product of pyrolysis of **2** in argon matrix with subsequent trapping in an argon matrix at 10 K. (a) IR spectrum of **1** computed at AE-CCSD(T)/cc-pCVTZ (unscaled). (b) IR difference spectra showing the photochemistry of **1** after irradiation with λ = 254 nm in argon at 10 K. Downward bands assigned to **1** disappear while upward bands assigned to **3** and 4 appear after 20 min irradiation time. (c) IR spectrum of *d*$_4$-**1** computed at AE-CCSD(T)/cc-pCVTZ (unscaled). (d) IR difference spectra showing the photochemistry of *d*$_4$-**1** after irradiation with λ = 254 nm in argon at 10 K. Bands pointing downwards assigned to *d*$_4$-**1** disappear and bands pointing upwards assigned *d*$_4$-**3** and *d*$_4$-**4** appear after 5 min irradiation time.

Table S1. Experimental (Ar matrix, 10 K) and computed IR frequencies of **1** and d_4-**1**, band origins in cm⁻¹, computed intensities (km mol⁻¹) in parentheses.

Mode	**1** Computed[a]	**1** Ar, 10 K[b]	d_4-**1** Computed[a]	d_4-**1** Ar, 10 K[b]	Symm	Assignment
18	3855 (61.3)	3643 (s)	2806 (37.3)	2689 (s)	A‘	OH str.
17	3840 (96.5)	3619 (s)	2795 (60.7)	2675 (s)	A‘	OH str.
16	3298 (2.0)	-	2459 (0.4)	-	A‘	CH_2 asym. str.
15	3195 (3.4)	-	2336 (17.8)	-	A‘	CH_2 sym. str.
14	1777 (292)	1712 (s)	1719 (290)	1668 (s)	A‘	C=C str.
13	1457 (11.2)	1403 (w)	1129 (27)	1112 (m)	A‘	CH_2 in-plane scissoring
12	1436 (117.6)	1377 (s)	1376 (205)	1315 (s)	A‘	C-O str.
11	1255 (86.2)	1218 (s)	973 (67)	959 (s)	A‘	OH def.
10	1207 (159.6)	1168 (s)	951 (61.7)	936 (s)	A‘	COH def.
9	983 (23.2)	961 (m)	871 (19.7)	853 (m)	A‘	CH_2 in-plane wagging
8	936 (49.8)	917 (m)	794 (1)	-	A“	C-O str.
7	727 (56.4)	730 (s)	669 (20.5)	661 (m)	A“	CH_2 out of pl. wagging
6	689 (51.8)	674 (m)	500 (21.2)	491 (m)	A“	CH_2 out-of-pl. rocking
5	652 (4.7)	658 (w)	547 (27)	546 (s)	A“	C-O out-of-pl. wagging
4	533 (24.7)	531 (w)	480 (24.9)	482 (m)	A‘	COC def.
3	451 (3.2)	429 (w)	379 (3.4)	-	A‘	CCO def.
2	363 (8.5)		268 (2.3)	-	A“	O-H out-of-pl. wagging
1	151 (184)		101 (98)	-	A“	O-H out-of-pl. wagging

[a]AE-CCSD(T)/cc-pCVTZ, harmonic approximation, unscaled frequencies, intensities (in parentheses) in km mol⁻¹. [b] Experiment: argon matrix, 10 K.; approximate relative intensities (w: weak, m: medium, s: strong).

References

[1] CFOUR, **2010**, p. http://www.cfour.de.

[2] a) J. Čížek, *J. Chem. Phys.* **1966**, *45*, 4256-4266; b) R. J. Bartlett, J. D. Watts, S. A. Kucharski, J. Noga, *Chem. Phys. Lett.* **1990**, *165*, 513-522; c) K. Raghavachari, *Annu. Rev. Phys. Chem.* **1991**, *42*, 615-642; d) J. F. Stanton, *Chem. Phys. Lett.* **1997**, *281*, 130-134.

[3] J. Dunning, Thom H., *J. Chem. Phys.* **1989**, *90*, 1007-1023.

[4] a) A. D. Becke, *Phys. Rev. A* **1988**, *38*, 3098-3100; b) A. D. Becke, J. *Chem. Phys.* **1993**, *98*, 5648-5652; c) C. Lee, W. Yang, R. G. Parr, *Phys. Rev. B* **1988**, *37*, 785-789.

[5] Gaussian09 Frisch, M. J. *et al.*, (Gaussian, Inc., Wallingford, 2013).

AE-CCSD(T)/cc-pCVTZ optimized structures (distances in bohr), electronic energies (in hartree) and zero-point vibrational energies (ZPVE).

3: Acetic acid *syn* (C_s)

C	2.74946139	–0.07890890	0.00000000
C	–0.07481134	0.19150104	0.00000000
O	–1.22944681	–2.09309915	0.00000000
O	–1.23550796	2.14952937	0.00000000
H	3.61306601	1.78008496	0.00000000
H	–3.02057985	–1.73668033	0.00000000
H	3.34076743	–1.13980424	–1.66145313
H	3.34076743	–1.13980424	1.66145313

E = –228.9697461
ZPVE = 38.9506 kcal mol^{-1}

3c: Acetic acid *anti* (C_s)

C	2.74067923	0.09758853	0.00000000
C	–0.09937231	–0.19887340	0.00000000
O	–1.41949616	2.00763396	0.00000000
O	–1.19562010	–2.17952706	0.00000000
H	–0.25272647	3.40055087	0.00000000
H	3.61552921	–1.75619826	0.00000000
H	3.34570052	1.14484861	1.66933842
H	3.34570052	1.14484861	–1.66933842

E = –228.9612832
ZPVE = 38.7852 kcal mol^{-1}

Enol **1** (C_s)

C	2.73083188	–0.18802690	0.00000000
C	0.21462218	–0.03141618	0.00000000
O	–1.17480999	2.13472715	0.00000000
O	–1.31524561	–2.06938716	0.00000000
H	–3.02480474	–1.45254194	0.00000000
H	–0.02733551	3.54144348	0.00000000
H	3.62851970	–2.01354000	0.00000000
H	3.87159304	1.50051666	0.00000000

E = –228.9256377
ZPVE = 38.3324 kcal mol^{-1}

Enol **1a** (C_{2v})

C	0.00000000	0.00000000	–2.72112041
C	0.00000000	0.00000000	–0.19027092
O	0.00000000	–2.05833678	1.31407511
O	0.00000000	2.05833678	1.31407511
H	0.00000000	3.52008546	0.23799806
H	0.00000000	–1.75310046	–3.76060477
H	0.00000000	1.75310046	–3.76060477
H	0.00000000	–3.52008546	0.23799806

E = –228.9241836
ZPVE = 38.5632 kcal mol^{-1}

Enol **1b** (C_2)

C	0.00000000	0.00000000	–2.73082874
C	0.00000000	0.00000000	–0.21856127
O	–2.16742127	0.07165086	1.18124782
O	2.16742127	–0.07165086	1.18124782
H	–1.76110103	–0.15757104	–3.73902281
H	1.76110103	0.15757104	–3.73902281
H	–1.96998543	–1.11025542	2.55070357
H	1.96998543	1.11025542	2.55070357

E = –228.9214503
ZPVE = 38.5361 kcal mol^{-1}

H_2O (C_{2v})

O	0.00000000	0.00000000	–0.12523284
H	0.00000000	–1.42414129	0.99376801
H	0.00000000	1.42414129	0.99376801

E = –76.3897971
ZPVE = 13.5150 kcal mol^{-1}

4: Ketene (C_{2v})

C	0.00000000	0.00000000	–2.44728093
C	0.00000000	0.00000000	0.03947148
O	0.00000000	0.00000000	2.23953625
H	0.00000000	–1.77777683	–3.43684544
H	0.00000000	1.77777683	–3.43684544

E = –152.5183135
ZPVE = 19.7893 kcal mol^{-1}

TS acetic acid rotation (C_1)

C	–0.04686928	0.22316486	0.01532142
C	2.69353122	–0.53068014	0.00253852
O	–1.67825209	–1.82479324	–0.11886397
O	–0.81398768	2.35002778	0.00844833
H	–2.01709182	–2.43140779	1.55913432
H	3.08897211	–1.75829529	1.60953779
H	3.88922915	1.13438128	0.07318352
H	3.07919387	–1.61899969	–1.70213447

E = –228.9493750
ZPVE = 37.8445 kcal mol^{-1}
υ_i = 564 cm^{-1}

TS-1: enol rotation 1–1a (C_1)

C	0.17418814	–0.08373298	–0.00140011
C	2.42179928	–1.22970229	0.00790018
O	–2.07165169	–1.33769634	–0.11452563
O	–0.22597777	2.45110346	0.01702151

H	4.15505849	–0.15434573	0.06721030
H	2.51363574	–3.26133369	–0.07338591
H	–2.50743968	–1.88466524	1.55891070
H	1.39381483	3.26861439	–0.08266921

E = –228.9187000
ZPVE = 38.1209 kcal mol^{-1}
υ_i = 388 cm^{-1}

TS-2 enol rotation **1a**–**1b** (C_1)

C	0.21761490	–0.00577061	0.00311189
C	2.71628601	–0.28291522	0.00303214
O	–0.93144697	2.29144975	–0.04068413
O	–1.45594368	–1.99252272	0.10844281
H	3.51809929	–2.15320452	–0.01007265
H	3.92513831	1.35604059	0.02064466
H	–2.66239148	2.00483291	0.43257499
H	–1.82467934	–2.51452522	–1.59168239

E = –228.9199633
ZPVE = 38.0401 kcal mol^{-1}
υ_i = 302 cm^{-1}

TS-3 enol **1** - acetic acid **3** (C_1)

C	–0.03154070	–0.08436602	–0.05576065
C	2.22901689	–1.51788810	0.06790924
O	0.44024335	2.30021562	0.01426939
O	–2.37835438	–0.91641700	–0.02842373
H	3.38158845	–1.25132858	–1.60882112
H	2.19712251	–3.42820395	0.79433562
H	–3.46813157	0.52161084	0.25577807
H	2.48367660	1.27379877	0.63869605

E = –228.8500606
ZPVE = 35.4580 kcal mol^{-1}
υ_i = 2181 cm^{-1}

TS-4 enol 1 –ketene (**4**) -H_2O elimination (C_1)

C	–2.85977545	–0.40969396	0.00719744
C	–0.44375384	0.28959677	–0.01491989
O	1.64505652	–1.83372442	–0.10691269
O	1.04795015	2.16882121	0.01850969
H	1.67058997	–2.68572937	1.50984290
H	2.59118696	0.12508984	0.02591875
H	–4.28588437	1.04209886	0.08591639
H	–3.38130539	–2.36971197	–0.12670844

E = –228.8504352
ZPVE = 35.0555 kcal mol^{-1}
υ_i = 1657 cm^{-1}

TS-5 enol **1a** acetic acid **3c** (C_1)

C	0.05039868	0.04528754	0.03912524
C	–2.09139006	1.69361026	–0.06170926
O	2.49446351	0.54820801	–0.00574700

O	–0.65035494	–2.26062402	–0.01074484
H	–3.14620921	1.65003005	1.69908061
H	–1.90963336	3.56022544	–0.88758049
H	–2.61219597	–1.07052170	–0.55811490
H	2.70243202	2.33279279	0.27725633

E = –228.8477326
ZPVE = 35.4655 kcal mol^{-1}
υ_i = 2135 cm^{-1}

Author Contributions: A. M. and P. R. S. conceived the idea. A. M. and A. K. E. carried out all experimental and computational studies. All authors analyzed and discussed all data. A. M. and P. R. S. co-wrote the manuscript. P. R. S. administrated the project.
